# Immunogenicity and Integration of a Decellularized Extracellular Matrix-Based Scaffold for the Reconstruction of Human Foreskin: A Preclinical Animal Study

**DOI:** 10.3390/bioengineering12111186

**Published:** 2025-10-31

**Authors:** Luca Pennasilico, Margherita Galosi, Lucia Biagini, Valentina Riccio, Caterina Di Bella, Federica Serino, Sara Sassaroli, Felice Ciabocco, Elena Bondioli, Giacomo Rossi, Vincenzo Aiello, Andrea Pratesi, Angela Palumbo Piccionello

**Affiliations:** 1 Futuravet, Veterinary Referral Center, 62929 Tolentino, Italy; 2 School of Bioscience and Veterinary Medicine, University of Camerino, 62024 Matelica, Italygiacomo.rossi@unicam.it (G.R.); angela.palumbo@unicam.it (A.P.P.); 3 Department of Translational Medicine and Clinical Science, University of Rome Tor Vergata, 00133 Roma, Italy; 4 Emilia Romagna Regional Skin Bank and Burn Centre Bufalini Hospital, AUSL Romagna, 47522 Cesena, Italy; 5 Foregen Onlus Association, 00141 Roma, Italy; 6 Independent Researcher, 35121 Padova, Italy

**Keywords:** extracellular matrix, foreskin dermal matrix, decellularized scaffold, regenerative medicine, tissue engineering, biocompatibility, immunogenicity, experimental study

## Abstract

**Simple Summary:**

This study evaluated the biocompatibility of a decellularized extracellular matrix (dECM) scaffold derived from human foreskin for potential use in foreskin reconstruction. Twenty-six rats were implanted with the scaffold in the hypodermal layer and divided into two groups: Group B (5-day follow-up) and Group A (30-day follow-up). Clinical monitoring assessed inflammation, and tissue samples were examined histologically and immunohistochemically for immune response, neovascularization, and recellularization. Only mild inflammatory signs were observed in the first days post-implantation, with slight neutrophil and eosinophil presence and moderate lymphocyte and macrophage infiltration. After 30 days, neovascularization and cell colonization were significantly higher compared to the 5-day group, while encapsulation remained mild and consistent across both groups. No evidence of severe immune reaction, necrosis, or cavity formation was detected. These results demonstrate that the foreskin-derived dECM scaffold elicited a limited immune response and promoted vascularization and cellular integration in vivo. The findings support its potential as a safe and effective biomaterial for foreskin reconstruction. Further research is needed to assess long-term integration and functionality before translation into clinical applications.

**Abstract:**

The reconstruction of the foreskin using autografts is a complex procedure. A novel decellularisation method for epithelial tissue has been developed, producing an extracellular matrix scaffold from human donor foreskin. This study evaluated the immune response and integration of this scaffold after implantation in rats, focusing on inflammatory infiltrate, neovascularization, recellularization, and foreign body reaction. Twenty-six rats underwent a 1 cm infrascapular incision with scaffold implantation in the hypodermal layer. Group A (13 rats) was subject to a 30-day follow-up period, while Group B (13 rats) was subject to a 5-day follow-up period. Inflammation at the implantation site was scored from 0 (none) to 3 (severe). Tissue explants were. After 5 days (Group B) and 30 days (Group A), a tissue explant was performed and examined histologically and immunohistochemically. The clinical evaluation revealed slight signs of inflammation during the initial five days following the implantation procedure. Neutrophil (0.87 ± 0.35; 1 ± 0.53) and eosinophil (0.61 ± 0.51; 0.75 ± 0.46) presence was slight, with no significant differences between groups. Lymphocyte infiltration was moderate (1.87 ± 0.35; 1.75 ± 0.46), exceeding macrophage presence (1.25 ± 0.46; 1.12 ± 0.35). Neovascularization and cellular colonization were significantly greater at 30 days (2 ± 0.53; 2.42 ± 0.53) compared to 5 days (0.57 ± 0.21; 0.62 ± 0.32). Encapsulation remained mild in all cases, with no intergroup differences (0.87 ± 0.35). These findings indicate that the decellularized extracellular matrix derived from human foreskin elicits minimal immune response while promoting neovascularization and cellular repopulation. This supports its potential use as a biocompatible scaffold in reconstructive procedures.

## 1. Introduction

Circumcision, the surgical removal of the foreskin, is one of the most common procedures worldwide, affecting about 38% of the global population [1,2]. Despite its prevalence, this practice generates much controversy, due to potential sexual consequences or psychological sequelae, especially in the case of infant and involuntary circumcision [3,4,5,6]. The loss of mechanoreceptors in the foreskin affects the ejaculation [7,8] while the lack of lubrication and roller-bearing action results in painful erection and intercourse [9]. In addition, early circumcised men show higher attachment insecurity and emotional instability, lower empathy and trust, higher sexual libido with unrestricted socio-sexuality, and higher stress and risk-taking attitudes [6,10].

The first efforts to restore the foreskin date back to Ancient Greek society [11]. Current approaches are either surgical or non-surgical [12,13]. Surgical techniques are associated with a range of complications, including edema, hematoma, flap necrosis, and protracted healing times, and are frequently associated with unsatisfactory results due to color and functional differences of the neo-prepuce [12,13].

To date, non-surgical methods are the prevailing approach for restoration of the foreskin. The division of these methods can be made according to the principle of taping versus tapeless, with the former being characterized by manual stretching and the latter by device-assisted tissue expansion. These techniques exploit mechanical stress applied to the penis skin, allowing for its lengthening and subsequent coverage of the glans over time. Non-surgical methods have yielded favorable results with minimal adverse effects; however, they are time-consuming, and the literature on these techniques is scant [13,14]. Although skin expansion results in complete glans coverage, with higher cosmetic appearance and functionality, there is no effective treatment for the restoration of the complex structure and specialized innervation of the foreskin. The foreskin is a complex mucocutaneous structure rich in vessels and nerve endings, consisting of inner mucosa, lamina propria, dartos muscle, dermis, and the outer epithelium. Thanks to its dense innervation and high concentration of mechanoreceptors, the prepuce is a very sensitive area involved in sexual pleasure [15]. Given these limitations, recent research has focused on decellularized extracellular matrix (dECM) scaffolds, which retain the native architecture and bioactivity of tissues while minimizing immunogenicity [16,17,18,19].

Decellularized ECM (dECM) biomaterials, produced by removing immunogenic components of donor tissues while preserving the native matrix, have shown promise in tissue repair and regeneration, known as acellular dermal matrix (ADM) [20]. The ECM is a large network consisting of extracellular macromolecules and minerals that surround, support, and give structure to cells and tissues in the body, including collagen, hyaluronic acid, proteoglycan, glycosaminoglycan, enzymes, glycoproteins, fibronectin, elastin, laminin, various cell growth factors, and hydroxyapatite. It is directly involved in tissue repair, provides physical support for cell adhesion, influences cell behaviors including migration, proliferation, and differentiation, and acts as a niche for stem cell differentiation [20,21]. dECM scaffolds proliferate and promote cell migration, proliferation, angiogenesis, and tissue remodeling following implantation [22].

After host implantation, there is an inflammatory phase followed by an increase in collagen levels and elastin production that promotes the neovascularization and deposition of new connective tissue. Subsequently, dECM biomaterials are gradually degraded and replaced by new host tissue. Inflammation and the specific cell types involved play a crucial role in the tissue repair phase. In this regard, macrophages and their polarization have been identified as fundamental in the evolution of certain pathological conditions [23,24] and in the tissue repair process [25,26].

Initially applied in burn and wound management, acellular dermal matrices (ADMs) are now widely used in breast reconstruction, hernia repair, and genitourinary surgery, offering benefits such as improved healing, vascularization, and resistance to infection [22,27,28,29,30].

Considering their biological origin, ADMs have an interesting application in the abdominal wall reconstruction of contaminated hernia to reduce erosion into the bowel wall, adhesion infection, and fistula formation [27,28,29]. In the genitourinary area, the acellular dermal matrix has been used in urethral reconstruction, bladder replacement, ureteral surgery, pubovaginalis sling, and hypospadias repair, with multiple benefits described [28]. However, ADMs also face limitations, including potential immune rejection, disease transmission, cost, and a lack of standardized protocols and high-quality clinical studies [27,31,32].

Based on the principles of “tissue engineering and regenerative medicine”, Purpura and colleagues [18] have developed a novel decellularized foreskin from human donors, which maintains the architecture and structural integrity of the dermal layer with a compact and well-preserved ECM. Cell viability is drastically reduced compared with samples of fresh frozen foreskin, reducing immunogenic power. In addition, the decellularized foreskin had a high level of fibroblast growth factor (FGFb), which is involved in regenerating a wide variety of tissue types, including skin, blood vessel, muscle, adipose, tendon, ligament, cartilage, bone, tooth, and nerve tissues. This novel dECM scaffold offers a very promising approach for the foreskin regeneration due to the presence of intrinsic anatomic and structural components that are biologically inherent in foreskin tissue [18].

This is the first in vivo study to assess the immunogenicity, vascularization, and cellular integration of a decellularized extracellular matrix scaffold derived from human foreskin. While previous research has described the decellularization process and characterized the resulting biomaterial in vitro, no studies have yet evaluated its biocompatibility within a living host. This work therefore provides the first preclinical validation of a foreskin-derived scaffold as a potential regenerative biomaterial for foreskin reconstruction.

The present study aims to assess the immunogenicity and cellular integration of a decellularized extracellular matrix scaffold derived from human foreskin, assessing inflammatory reaction, neo-vascularization, recellularization, and potential foreign body reaction. Our hypothesis is that this new decellularisation method effectively removes antigenic components while preserving the extracellular matrix structure, thereby promoting fibroblast recellularization in an animal model. While previous research has described the decellularization process and characterized the resulting biomaterial in vitro, no studies have yet evaluated its biocompatibility within a living host. This work therefore provides the first preclinical validation of a foreskin-derived scaffold as a potential regenerative biomaterial for foreskin reconstruction.

## 2. Materials and Methods

### 2.1. Study Design

This study involved 26 Wistar male rats weighing approximately 350 g and 4 months of age, acquired from Charles River Laboratories Italia S.r.l. (Calco, Italy). The subjects were housed in separate cages (two rats for each cage) with an artificial day–night cycle (12 h light/dark) and constant temperature (20–22 °C) and humidity (45–55%). The rats were randomly divided into two groups (A group = 13 rats; B group = 13 rats). A group was involved in the study for 30 days, while the B group was involved for 5 days to evaluate two different stages of host inflammatory and immune response. This study was conducted at the University of Camerino and approved by the Ministry of Health with protocol number 424/2021-PR. All experiments were performed in accordance with the ARRIVE guidelines. To enable comparison of the histomorphology and immunohistochemical results, skin samples were taken from the infrascapular region of five rats suppressed as part of a health monitoring plan at the laboratory animal breeding facility of the University of Camerino.

### 2.2. Decellularization Protocol

The scaffold was produced by Purpura and colleagues [18] at the Emilia Romagna Regional Skin Bank. Briefly, the foreskin was harvested from adult human donors for therapeutic purposes. The harvest foreskin was placed in RPMI 1640 medium (Biowest, Riverside, MO, USA), antibiotics, and 10% cryoprotectant (CRYO·ON DMSO; Alchimia, Ponte San Nicolò, Italy) and frozen at −80 °C.

The foreskin was decellularized with a combination of physical and enzymatic methods. The epidermal and dermal layers were separated with 2.5% trypsin, diluted to 1× (Life Technology, Monza, Italy) with 0.9% NaCl saline solution (Fresenius Kabi AG, Bad Homburg, Germany). The dermal tissue was treated with 2.5% trypsin, diluted to 4× (Life Technology, Monza, Italy) with 0.9% NaCl saline solution, and incubated in a temperature- and atmosphere-controlled environment for 24 h. At the end of the procedure, the samples were washed in 0.9% NaCl saline solution. The decellularized foreskin was immersed in RPMI medium with 10,000 IU/mL penicillin, 10 mg/mL streptomycin, and 25 μg/mL amphotericin B for 15 min and stored in liquid nitrogen.

The method employed involves the utilization of an enzyme, trypsin, for the purpose of eradicating the cellular component and DNA present, without inducing toxicity. Cells exposed to diverse concentrations of enzymes initially disengage from the tissue matrix, subsequently undergo necrosis, and ultimately result in complete cell removal.

### 2.3. Characteristics of Decellularized Foreskin

**Figure 1:**
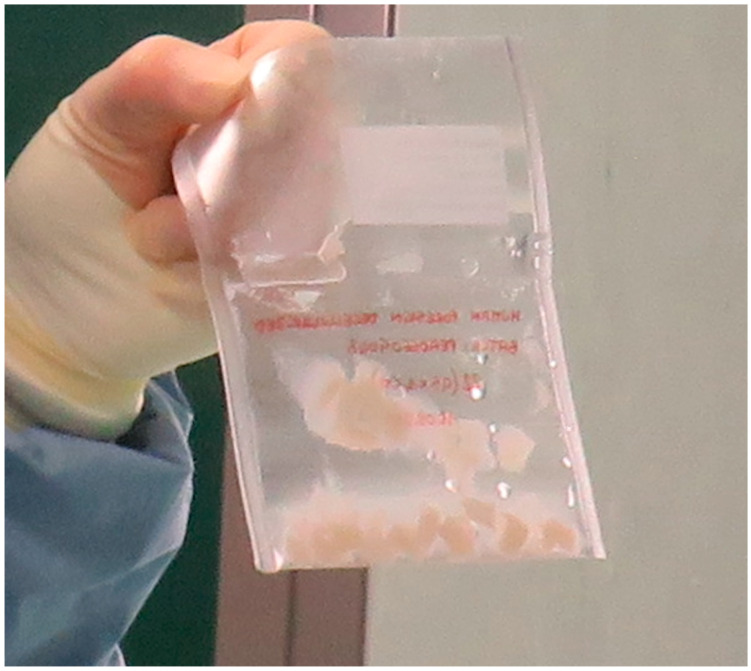
Sample of foreskin after decellularization. The white coloration of the scaffold indicated the removal of the epidermal layer. The foreskin was cut into 20 mm × 20 mm segments before subcutaneous implantation in rats.

The properties of this biomaterial were evaluated and described by Purpura et al. [18]. The macroscopic assessment showed white coloration of the decellularized tissue, indicating the removal of the epidermal layer while preserving the extracellular matrix with bioactive components (Figure 1). Microbiological analysis confirmed the absence of bacteria and fungi during all stages. The spectrophotometry and qualitative histological analysis of the samples showed a drastic reduction in cellular viability. In addition, histological analysis (H&E and Masson’s Trichrome staining) showed maintenance in the architecture and structural integrity of the dermal layer with the presence of well-maintained collagen fibers in the decellularized foreskin samples compared with those of fresh frozen foreskin. An ELISA assay demonstrated that the FGFb content doubled after decellularizing the foreskin, corresponding to tissue bioactivity. Finally, regarding mechanical tensile properties, there were no differences in maximum load, tensile strength, Young’s modulus, or stiffness between the decellularized and fresh frozen foreskin.

### 2.4. Surgical Procedure

**Figure 2:**
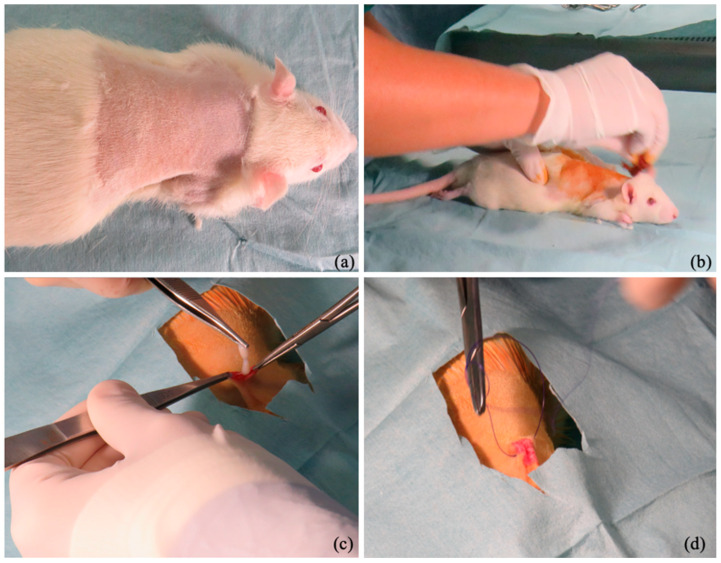
(**a**,**b**) Surgical site skin preparation. (**c**,**d**) Intraoperative picture shows the implantation of decellularized foreskin in the hypodermal (**c**) layer and skin suture (**d**).

After the subcutaneous administration of buprenorphine (50 mcg/kg; Buprefelican, Dechra S.r.l., Torino, Italy) and carprofen (5 mg/kg; Rymadil, Zoetis S.r.l., Roma, Italy), all rats underwent general anesthesia through the intraperitoneal administration of ketamine (70 mg/kg; Lobotor, Acme S.r.l., Cavriago, Italy) and xylazine (10 mg/kg; Sedaxylan, Dechra S.r.l., Torino, Italy). Furthermore, additional oxygenation was ensured with the administration of pure oxygen via a face mask. When a good anesthesiologic plan was achieved, the subjects were positioned in sternal recumbency, and a square infrascapular area of about 6 cm^2^ was clipped. Preoperative skin preparation was performed with povidone–iodine (10%) and ethyl alcohol (95%). Using aseptic techniques, an infrascapular skin incision of about 1 cm was made, and a decellularized extracellular matrix scaffold (about 20 mm in diameter) was implanted in the hypodermal layer (Figure 2). After implanting the scaffold, the skin was sutured using a USP 4/0 polydioxanone thread (Ethicon, Somerville, NJ, USA). The rats were warmed using an infrared lamp during the awakening phase. Heating support was discontinued when the rectal temperature was above 37.5 °C. Other physiological parameters monitored were the heart and respiratory rate, and the degree of sedation until the rats were fully awake. The Rat Grimace Scale was used to evaluate the pain degree and discomfort in the postoperative period. The rats received subcutaneous administration of buprenorphine (50 mcg/kg; Buprefelican, Dechra S.r.l., Torino, Italy) and carprofen (5 mg/kg; Rymadil, Zoetis S.r.l., Roma, Italy) every 24 h for 2 days and enrofloxacin (0.2 mg/mL; Elanco Italia S.p.a., Sesto Fiorentino, Italy) dissolved in drinking water for 5 days. During the first five postoperative days, the subjects underwent daily clinical examinations to assess their health status and physiological functions.

### 2.5. Clinical Evaluation

The infrascapular region (implant site) was clinically monitored to detect the key signs of inflammation. Clinical evaluations of all subjects (both B and A groups) were performed daily (T0, T1, T2, T3, T4, and T5) for the first 5 days postop and subsequently every 5 days (T10, T15, T20, T25, T30) until 30 days postop, but only in the A group. Specifically, heat (calor), redness (rubor), and swelling (tumor) were assessed using a 4-point score, ranging from 0 (no signs) to 3 (severe signs of inflammation). The scoring system was applied:

- Calor: a score of 0 indicated that the temperature of the infrascapular region is the same as the body skin, a 1 indicated a slight increase in temperature, a 2 indicated a moderate increase in temperature, and a 3 indicated a significant increase in temperature. The score was established to palpate these regions.
- Rubor: the absence of infrascapular skin redness was classified as 0, a slight reddening of the skin at the surgical site was classified as 1, reddening of the skin not exceeding two millimeters was classified as 2, and reddening of the skin at the surgical site exceeding two millimeters was classified as 3.
- Tumor: increased infrascapular skin thickness of less than 2 mm compared with the preoperative thickness was rated as 0, an increase between 2 and 4 mm was 1, an increase between 4 and 6 mm was 2, and an increase of more than 6 mm was 3. The skin measurement was performed using a manual caliper

### 2.6. Histological Analysis

The subjects were euthanized at 5 days (B group) and 30 days (A group) post-implantation of the scaffold. The same surgical procedure was applied to animals belonging to the control group in order to obtain control tissue samples for subsequent histological analysis. All rats underwent general anesthesia via the intraperitoneal administration of ketamine (70 mcg/kg; Lobotor, Acme S.r.l., Cavriago, Italy) and xylazine (10 mg/ kg; Sedaxylan, Dechra S.r.l., Torino, Italy). Lastly, euthanasia was performed through carbon dioxide (CO_2_) inhalation. A skin infrascapular biopsy of about 6 cm in diameter centered on the skin scar was obtained. The biopsies were fixed in 10% buffered formalin and then routinely processed. The samples were dehydrated through a graded series of alcohol and cleared with an ethanol/xylene solution before being embedded in paraffin wax. Serial sections (3 μm thick) were cut, dewaxed, and stained with Hematoxylin–Eosin; after dehydration, they were mounted for observation under an optical microscope (Leica DM2500, Wetzlar, Germany) or placed on polarized slides for immunohistochemical staining. Histological and immunohistochemical assessment was performed blindly by a single operating pathologist. For each sample, five microscopic fields were evaluated using a ×40 objective, ×10 eyepiece, and a square eyepiece graticule (10 × 10 squares, with a total area of 62,500 μm^2^). The histological examination of the decellularized foreskin matrix included an assessment of neutrophils, eosinophils, lymphocytes, macrophages, and cell colonization (fibroblasts). A 0–3 scoring system was applied as follows: 0 = 0 to 3 inflammatory cells per field; 1 = 3 to 5 cells per field; 2 = 5 to 15 cells per field; 3 = 15 cells per field.

### 2.7. Immunohistochemical Analysis

Immunohistochemistry was performed using the avidin–biotin–peroxidase complex method (Vectastain Elite ABC Kit; Vector Laboratories, Newark, CA, USA) with 3,3′-diaminobenzidine (Vector Laboratories) as chromogen [33]. Sections were deparaffinized, and endogenous peroxidases were inhibited with 0.3% hydrogen peroxide in methanol for 30 min, while antigen retrieval was achieved with heat microwave treatment in citrate buffer, pH 6. Non-specific binding sites were blocked by incubating sections at room temperature for 1 h with normal serum, obtained from the species corresponding to the secondary antibody, and diluted in a solution of 1% bovine serum albumin, 1% polyvinylpyrrolidone, and 1% tris-buffered saline (BSA-PVP-TBS). Then, sections were rinsed and incubated with the specific secondary antibody at room temperature for 45 min, followed by the avidin–biotin–peroxidase complex at room temperature for 45 min. Finally, the reaction was revealed with diaminobenzidine (DAB) for 3 min and blocked with sterile water. Sections were counterstained for 15 s with Harris’s hematoxylin, dehydrated, and mounted. All slides were tested with mono (mAbs) and polyclonal (pAbs) primary antibodies. Specifically, mouse mAb anti-vimentin (dilution 1:100, Clone V9, M0725, Dako, Denmark) was used to evaluate the fibroblastic reaction, forming a capsule surrounding the scaffold, and rabbit pAb anti-Factor VIII (Dilution 1:200, RP012-05, CliniSciences, Guidonia, Italy) was used as a marker of neovascularization. To characterize the macrophagic immune response, the ionized calcium-binding protein molecule 1 antibody (Iba1, dilution 1:300, 019-19741, FUJIFILM, Wako, Neuss, Germany), a pan-macrophage marker, was used to evaluate the total number of macrophages in tissues, while anti-macrophage scavenger receptor (MSR-A; CD204, dilution 1:100, KAL_KT022, Transgenic Inc., Chuo-ku, Kobe, Japan) and inducible Nitric Oxide Synthase antibody (iNOS, Ab3523, Abcam, Cambridge, MA, USA) were used to identify, respectively, the M1 and M2 macrophage phenotypes. For Vimentin and anti-Factor-VII, the semi-quantitative assessment regarding the immunohistochemical reaction in the antigens was scored as follows: 0 (absence of antigen expression), 1 (weak and spotted antigen expression), 2 (weak but profuse antigen expression across the whole specimen), and 3 (profuse and marked antigen expression). Iba-1+ cells were counted in five fields surrounding the scaffold at 40X magnification, and the results were expressed with a score of 0–3, as previously described for histological analysis. For CD204 and iNOS markers, the number of M2 or M1 polarized cells is presented as a percentage.

### 2.8. Statistical Analysis

A sample size calculation was performed considering previous data for rats (Prudente et al., 2016) [34]. Power calculation was conducted for a two-tailed *t*-test with a power of 0.95, an alpha error of 0.05, and an effect size of 1.36 (G*Power Version 3.1.9.3). This suggested that a minimum of 13 rats per group was sufficient to detect significant differences (G*Power v. 3.0.10; University of Düsseldorf, Düsseldorf, Germany) [35]. Statistical analysis was performed using MedCalc 9.0 (MedCalc version 9.2.10). All data resulted in normal distributions according to the Shapiro–Wilk test. Clinical parameters were evaluated with the one-way ANOVA (analysis of variance) test to perform a comparison between times. Moreover, to compare the two groups, histological and immunohistochemical data were analyzed using the Kruskal–Wallis test, followed by the Dunn post hoc test. All results are presented as the mean ± standard deviation. Differences with a *p*-value < 0.05 were considered statistically significant.

The GraphPad Prism 10 statistical software for macOS, version 10.1.1-270 (GraphPad Software Inc., San Diego, CA, USA) was used to produce graphs.

## 3. Results

### 3.1. Clinical Outcomes

**Figure 3:**
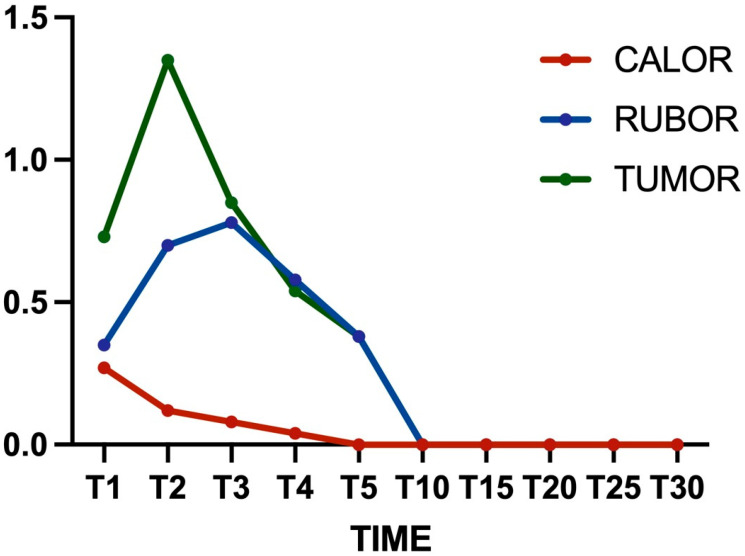
Graphical representation of calor, rubor, and tumor scores at different times during the study. The score (0–3) is indicated on the y-axis, and the time since the surgery is on the x-axis. The tumor score changed moderately 2 days post-implantation but decreased in the follow-ups (green line).

All animals were included in the analysis. No signs of infections or other injuries (such as scratch lesions) were identified in any subjects. Overall, no subjects showed severe clinical signs of inflammation. The main alterations were recorded in the first five days post-implantation (Table 1); after this period, all scores were 0 for the remaining 25 days (Figure 3).

### 3.2. Host Inflammatory and Immune Response to Scaffold

**Figure 4:**
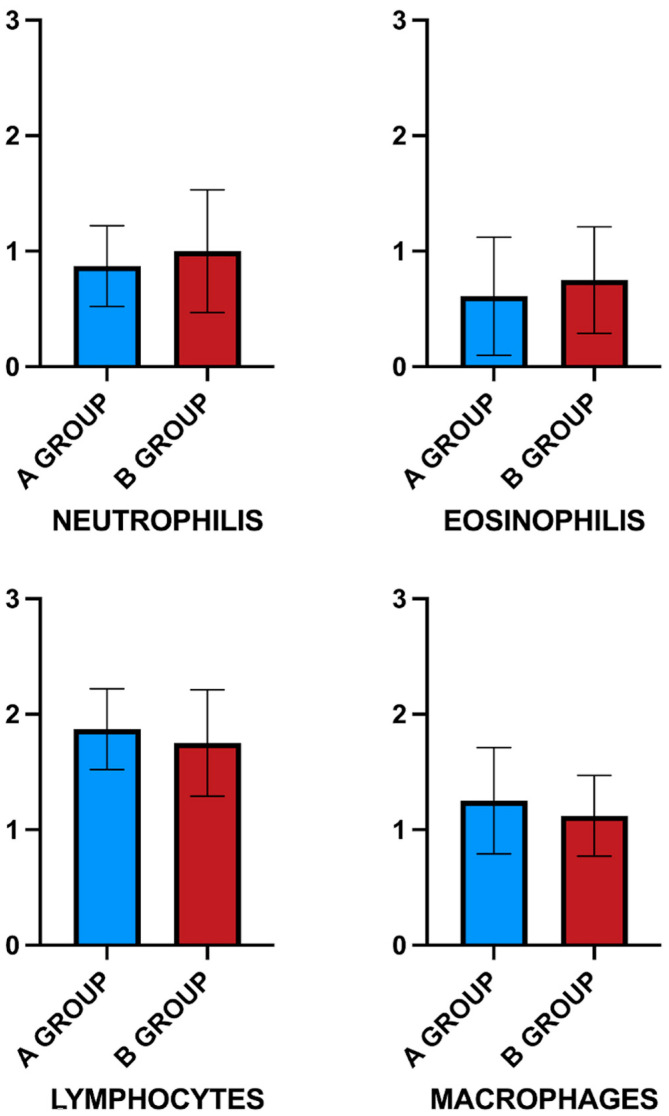
Graphical representation of inflammatory cell infiltrates that appear similar between the two periods of the study. A moderate lymphocyte response was observed in the A and B groups.

To better evaluate the inflammatory and immune responses within the scaffold, the inflammatory cell subtypes were graded based on a 0–3 scoring system (corresponding to absent, mild, moderate, and severe inflammation) using cell counts. The data showed a mild acute inflammatory response after decellularized foreskin implantation. Specifically, the presence of neutrophils and eosinophils was slight, without any statistical difference between the A and B groups (*p* > 0.05). Conversely, a moderate degree of adaptive immune response was observed. In particular, the presence of lymphocytes was moderate and higher than that of macrophages in both groups. Instead, the comparison between the two groups showed no statistical differences (*p* > 0.05). Conversely, the cutis of animals in the control group (C) exhibited minimal evidence of inflammatory cells, particularly in the subcutaneous layer. The inflammatory cell population was similar at 30 days post-implantation with a mainly adaptive immune response (Figure 4) (Table 2).

### 3.3. Macrophage Polarization

**Figure 5:**
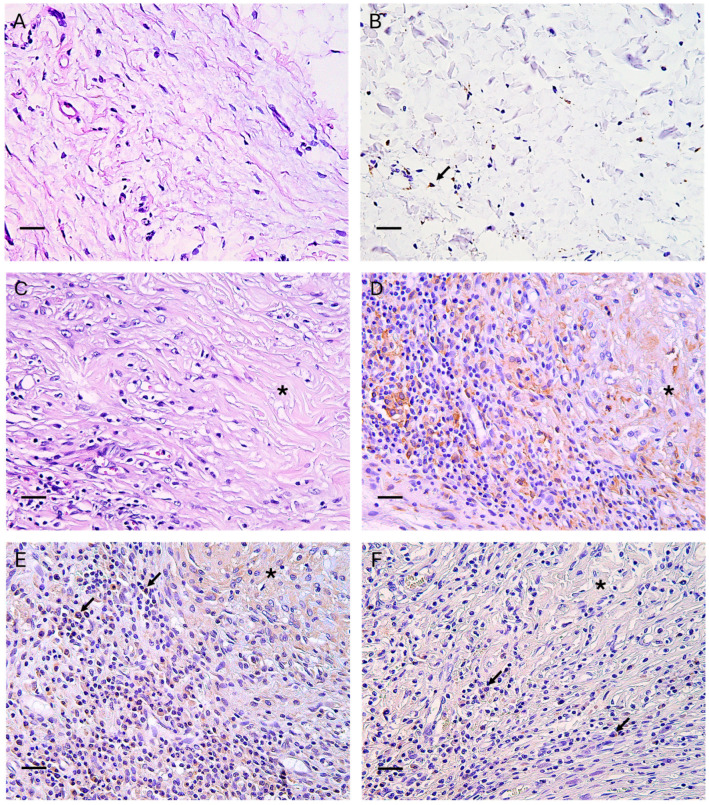
Histological aspect (**A**) and Iba-1 immunohistochemical labeling (**B**) of healthy skin from the control group. Few resident macrophages are labeled with the Iba-1 marker. (**C**) Histological aspect of the implanted scaffold (asterisk) and surrounding tissues at 30 days post-implantation. The scaffold is surrounded by a diffuse inflammatory reaction; macrophages and eosinophils are visible (H&E stain, scale bar = 50 μm). (**D**) Immunohistochemistry reveals many Iba-1+ macrophages, frequently located in perivascular areas. (**E**) In a large percentage, Iba-1+ macrophages are also positive for iNOS (black arrows), indicating prevalent M1 polarization. (**F**) A few macrophages show CD204 positivity (black arrows), a typical marker of M2 differentiation. (**B**–**F**), IHC stain, Harris’s hematoxylin nuclear counterstain; scale bars = 50 µm.

To establish the macrophages’ predominant phenotype, the percentage of iNOS-positive macrophages characteristic of M1 polarization and CD204-positive macrophages typical of the M2 phenotype was calculated and compared with the total number of Iba-1+ macrophages. The immunohistochemical count of the total IBA-1 positive macrophages confirmed the mild–moderate presence of macrophages inside the scaffold and in the perivascular area in both groups (Figure 5B), while, in the control group, macrophage colonization was considered as absent (score 0 = 0 to 3 inflammatory cells per field) (Figure 5). All counts were performed in the same microscopic fields for each of the three markers and consequently expressed as a percentage value. Five days post-implantation, 87% of macrophages showed the M1 phenotype, and only 13% of the total macrophages presented the M2 phenotype. During the last sampling, a slight increase in the M2 phenotype was registered (M1 = 75%; M2 = 24%), without statistical differences between the groups (*p* > 0.05).

### 3.4. Neo-Angiogenesis, Fibroblastic Colonization, and Capsule Formation

In order to ascertain the extent of neovascularization within the scaffold, the expression of Factor VIII was evaluated. This factor is typically expressed in the vessels present for tissue vascularisation by arteries, veins, and capillaries. Consequently, the focus was shifted to the newly formed vessels surrounding the scaffold.

Specifically, neovascularization was moderate, with the presence of medium (10–20 µm) vessels in deep portions of the biomaterial at 30 days. Conversely, neo-angiogenesis was significantly poor at 5 days and characterized by spotted microvessels (5–10 µm) within the matrix. Similarly, cell colonization was statistically significant in the A group (*p* < 0.05).

**Figure 6:**
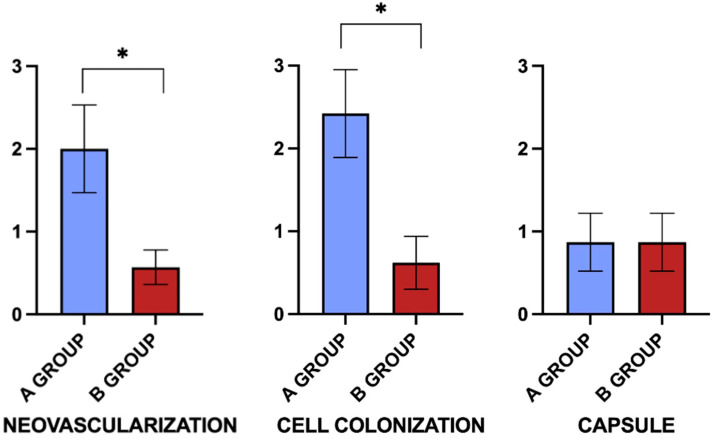
Graphical representation of data related to scaffold integration at the two different timepoints of the study. * *p* < 0.05, differences compared with the B group.

**Figure 7:**
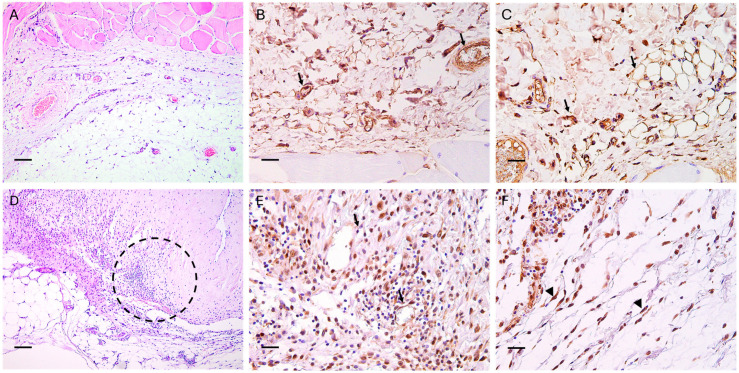
(**A**) Histological presentation of healthy skin. (**B**) Normal expression of anti-Factor VIII antibody by arteries and capillaries (black arrows) and (**C**) of anti-vimentin antibody by mesenchymal cells in the tissue, vessels, and hepatocytes membrane (black arrows). (**D**) Histological presentation of the scaffold surrounded by moderate inflammatory reactions and cell proliferation 30 days post-implantation (H&E stain, scale bar = 100 μm). (**E**,**F**) Higher magnification of the hypodermis–scaffold interface showing cell proliferation and infiltration (circle). Slight neo-angiogenesis is evident (black arrows) after immunohistochemical staining with anti-Factor VIII antibody (black arrows) (**E**). Anti-Vimentin-stained tissues show multiple cells with marked labeling (arrowheads), indicating fibroblastic proliferation (**F**). The abundant cellularization (circle) (**D**), microvessels (**E**), and fibroblasts (**F**) are not associated with a capsule or a net demarcation between the scaffold and the hypodermis, indicating the overall integration of the scaffold and not a foreign body reaction. IHC staining, Harris’s hematoxylin nuclear counterstain; scale bars = 50 µm.

**Figure 8:**
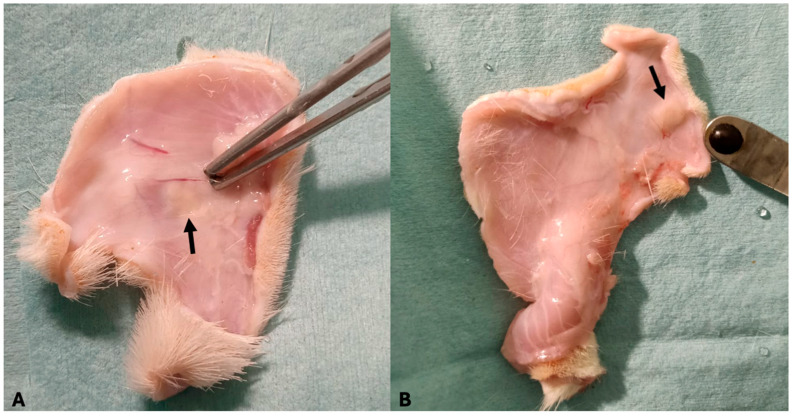
(**A**,**B**) Skin biopsy performed after 30 days on two rats. The presence of a capsule or cavity formation around the decellularized scaffold (black arrow) was absent.

The foreign body reaction is often characterized by a fibrotic response, evaluated with anti-vimentin antibodies. The diffusion of positively stained fibroblasts forming a capsule surrounding the scaffold was significantly high at 30 days post-implantation. Encapsulation was mild in all subjects, without differences between the two groups (Table 3) (Figure 6 and Figure 7). In addition, cavity formation and necrosis were absent in both groups (Figure 8).

## 4. Discussion

Foreskin restoration remains a challenge for regenerative medicine and tissue engineering due to the complexity of its vascularization and nerve networks. In recent years, a few attempts have been made to develop a biomaterial that can accurately reproduce the peculiar intrinsic structure of the foreskin. To the best of our knowledge, only three studies have described a decellularized extracellular matrix scaffold derived from human foreskin [16,17,18]. Among these decellularized foreskins, Purpura et al. [18] demonstrated that, in vitro, the decellularization of human foreskin with a combined physical–enzymatic process eliminates antigenicity and preserves the mechanical properties of the tissue, similar to fresh foreskin samples. Nevertheless, no studies have evaluated the biocompatibility of decellularized extracellular membranes from human foreskin donors in vivo. The immunogenicity of the scaffold is a crucial point for its application in the clinical field [36]. The goal of decellularization treatment is to eliminate the immunogenicity of tissue while preserving the structural components of the extracellular matrix. However, complete cellular removal is difficult to achieve, and excessively aggressive treatments may result in inherent damage to components of the extracellular matrix [37].

### 4.1. Acute Immune Response to Biomaterial Implant in the Rat

We evaluated the acute inflammatory infiltrate at 5 days post-scaffold implant in the host. Hyperacute or acute rejection of a biomaterial can occur within minutes or weeks post-implantation, which is essentially mediated by neutrophils in the early stage (24–48 h), followed by eosinophils, mast cells, macrophages, and B and T cells [38,39]. The acute inflammatory response is a physiological event that occurs after scaffold implantation; however, the degree and duration are related to the type of scaffold material [40,41]. Slight acute inflammation is required to start the process of tissue regeneration, and the normal restoration of injured tissues needs to be controlled this way [42]. In the control group, however, the inflammatory cell component is primarily made up of resident tissue cells, such as macrophages. These cells are usually present in small numbers in healthy tissue to recognize and present antigens.

In this study, no signs of severe acute inflammatory response were detected. The neutrophilic infiltration around the scaffold was slight at both 5 and 30 days post-implantation. According to Macleod et al., the absence of intense acute inflammation in the first days suggests a degree of tolerance to this scaffold. Conversely, the prolonged and excessive presence of neutrophils can lead to fibrotic encapsulation and the consequent failure of scaffold integration [43]. In our study, the presence of eosinophils was slight in both timepoints. The role of eosinophils in the immune response after dECM implantation is not well described. Generally, the presence of an eosinophilic infiltrate may represent a foreign body reaction [40]. By contrast, two studies concerning the use of an acellular matrix to treat cardiac and nerve injuries have shown that eosinophils promote tissue repair [44,45]. The eosinophilic response may be influenced by the anatomic region; for example, it can be stronger in the subcutaneous layer if compared with other anatomical sites [46]. Furthermore, massive eosinophilic infiltration indicates a severe acute inflammatory response [37].

### 4.2. Chronic Immune Response to Biomaterial Implant in the Rat

Following the acute inflammatory response that occurs in the first few days after surgery, the chronic inflammatory response takes place, mediated by macrophages, lymphocytes, and plasma cells [1]. Chronic inflammation may damage the scaffold and elicit a foreign body reaction [47,48]. However, a minimal chronic reaction surrounding the scaffold supports its integration and longevity [40]. In our study, we found a slight–moderate innate immune response mainly mediated by macrophages. These cells are considered the most relevant due to their constant role during the chronic response to biomaterials [49]. The hematoxylin and eosin stain showed the mild presence of macrophages around the scaffold, which was confirmed by the IBA-1 positivity. Re-cellularization in transplanted scaffolds can significantly inhibit leukocyte infiltration, which inhibits ECM destruction and injury of the graft post-operation [50]. Macrophage phenotyping is considered a good indicator of implanted scaffold biocompatibility. The M1 macrophage phenotype produces IL-1β, IL-6, and TNF-α, which are recognized as pro-inflammatory cytokines. Although the M1 polarization of macrophages is characteristic of the attack–rejection phases of the scaffold, and the immunophenotype that favors integration is considered the M2, we observed an initial inflammation characterized by a robust M1 response [49]. This phenotype is associated with an acute inflammatory reaction and is important for effective neo-angiogenesis and the subsequent integration of the scaffold [51]. Therefore, the differentiation of macrophages into M2 is important but must occur later, in the more advanced stages of the integration–repair process. In fact, M2 macrophage and Th2 cells are typical of a regenerative tissue response [52]. Implanting decellularized scaffolds can encourage the polarization of macrophages toward a constructive phenotype, first M1, and then M2. After colonization, neo-formed mesenchymal cells (MCs) suppress the proliferation of T cells, regulate the Th1/Th2 ratio, control the functions of regulatory T cells (Tregs), and secrete interleukins—particularly IL-10 and TGF-β [53]. Additionally, MCs increase the expression of Matrix Metalloproteinase (MMP)-1, MMP-3, and MMP-13 and maintain the remodeling process of the ECM [49,54,55]. However, macrophage polarization can be conditioned by the native tissue of the extracellular matrix-derived scaffold, particularly because the acellular dermal matrix promotes M1 polarization [56]. Conversely, the adaptive response elicited by lymphocytes was moderate at both timepoints of the study, possibly indicating the presence of some residual foreign epitopes in the scaffold.

### 4.3. Characterization of the In Vivo Host Remodeling Response

Our results showed a good remodeling of the scaffold 30 days post-implantation. The revascularization of biomaterials is essential for their survival and integration; nevertheless, accelerated angiogenesis may correlate with increased foreign body reactions. In our model, it is interesting to observe that—similar to what was observed in vivo by Xu et al. in a model of acellular dermal matrix for the abdominal wall repair—the neovascularization of the scaffold was moderate after 30 days of implantation (A group) but slight after 5 days (B group) [57].

Another key event for integrating a scaffold is its recellularization in the host. This process begins with inflammatory cell migration and the following revascularization that enables recellularization [58]. In our study, the fibroblastic colonization of the scaffold was massive 30 days post-implantation, assuming properties of the surrounding host tissue (dermis). Conversely, the presence of fibroblasts surrounding the scaffold was minimal at both times of the study. This meant that the host’s fibroblasts infiltrated the scaffold to guarantee its integration in the tissue and did not form a capsule, typically present in foreign body reactions [59].

Clinical evaluations allowed us to exclude inflammatory and immune reactions associated with other factors, such as infection of the surgical site or traumatic injuries during the postoperative period.

### 4.4. Animal Model

Rats were chosen as a model of immunogenicity in a preclinical assessment of this scaffold. As previously described, the subcutaneous surgical rat model is widely used to evaluate the biocompatibility of decellularized scaffolds derived from different tissues.

In our case, the biomaterial was placed in the subcutis without any suturing, as suggested by numerous studies. The suture method of implantation may increase the inflammatory reaction associated with suture materials. In immunogenicity studies, it is crucial to use a technique that minimizes the inflammatory infiltrate and promotes the recellularization of the scaffold to obtain more objective outcomes.

The rat represents a common, cost-effective, and reliable model to assess the immune response in vivo before the application of a scaffold in large animal models [60,61].

### 4.5. Limitations of the Study

This study had several limitations. Firstly, the absence of a control group hinders the ability to compare the effects of the surgical procedure over the course of the monitoring period. This bias means we cannot distinguish whether the immune response is associated with the scaffold or intrinsic host factors. Without a positive or negative control group, we could not establish for sure that the inflammatory infiltrate is a direct consequence of the scaffold’s characteristics. However, control animals were included for the evaluation of the immunohistochemical expression of the markers used in the skin of the infrascapular region of healthy subjects.

Except for macrophages, this study lacks a complete characterization of the phenotypical differentiation of the inflammatory cells involved in the immune reaction to the scaffold. This is a crucial element for obtaining more detailed information on the positive or negative reaction to scaffold integration. Additionally, analyzing cytokines is considered the most accurate method for evaluating the inflammatory environment, given their influence on immune cell activity [49]. Regarding macrophage differentiation, a limited number of markers were selected to evaluate the macrophage phenotypes (Iba-1, iNOS, and CD204) that may not fully capture them. These offer a general overview of the macrophage immunological response, but they could be implemented in conjunction with a more in-depth study of the extracellular matrix or collagen composition. Such an approach would be well-suited to certain histopathological studies. However, when more specific methods such as flow cytometry are unavailable, immunohistochemistry is an effective and inexpensive way to study cellular expression in formalin-fixed paraffin-embedded tissue.

Another limitation is the lack of an assessment of scaffold degradability. dECM degradation plays a key role in recruiting progenitor cells to sites of tissue remodeling. The absence of any biomaterial degradation can prevent cellular activity and colonization. Conversely, expedited degradation not supported by the deposition of new tissue can lead to encapsulation of the scaffold. The stability and clinical efficacy of a decellularized extracellular matrix depend on the balance between implant degradation and neo-tissue formation [57,62].

Our macroscopic results showed that the size of the scaffold was mildly reduced in the second phase of the study.

The final limitation is the relatively short duration of the study. A more protracted evaluation is needed to identify the possibility of chronic rejection [61,62].

## 5. Conclusions

For the first time, we evaluated the in vivo biocompatibility of a decellularized extracellular scaffold derived from human foreskin in a rat model. The host response and tissue remodeling were evaluated histologically and immunohistochemically in 26 rats.

Outcomes showed mild acute inflammatory infiltrate at both the time of study (5 and 30 days). Conversely, the adaptive immune response mediated by lymphocytes was moderate. The presence of macrophages was mild, and M1 macrophages represented the predominant phenotype at 5 and 30 days. The formation of new vessels and recellularization within the scaffold was abundant after 30 days. Additionally, no signs of foreign body reaction (necrosis, capsule, and cavity formation) were detected.

Overall, our results showed that decellularized extracellular matrix derived from human foreskin did not evoke a significant immune response, supporting neovascularization and cell colonization when implanted in a host. Furthermore, the long-term host response should be evaluated.

This scaffold could be promising for reconstructing human foreskin, due to its low immunogenicity; however, further studies in large animal models are needed to evaluate its real vascularization, innervation, and engraftment when implanted in the genital region before clinical applications in humans.

## Figures and Tables

**Table 1:** Mean ± standard deviation of clinical parameters. * *p* < 0.05, differences compared with T1. # *p* < 0.05, differences compared with T2. ° *p* < 0.05, differences compared with T3.

	T1	T2	T3	T4	T5
Calor	0.27 ± 0.10	0.12 ± 0.05	0.08 ± 0.01 *	0.04 ± 0.01 *^#^	0 *^#^
Rubor	0.35 ± 0.18	0.70 ± 0.30 *	0.78 ± 0.25 *	0.58 ± 0.14	0.38 ± 0.19 ^#^°
Tumor	0.73 ± 0.23	1.35 ± 0.44 *	0.85 ± 0.30 ^#^	0.54 ± 0.12 ^#^	0.38 ± 0.09 *^#^°

**Table 2:** Mean ± standard deviation of inflammatory cells histological scoring data. ^#^ *p* < 0.05, differences compared with the C group.

	A Group	B Group	C Group
Neutrophils	0.87 ± 0.35 ^#^	1 ± 0.53 ^#^	0.2 ± 0.44
Eosinophils	0.61 ± 0.51 ^#^	0.75 ± 0.46 ^#^	0
Lymphocytes	1.87 ± 0.35 ^#^	1.75 ± 0.46 ^#^	0.2 ± 0.44
Macrophages	1.25 ± 0.46 ^#^	1.12 ± 0.35 ^#^	0.2 ± 0.44

**Table 3:** Mean ± standard deviation of histological and immunohistochemical parameters. * *p* < 0.05, differences compared with the B group. ^#^ *p* < 0.05, differences compared with the C group.

	A Group	B Group	C Group
Neovascularization	2 ± 0.53 *^#^	0.57 ± 0.21 ^#^	0.2 ± 0.44
Cell colonization	2.42 ± 0.53 *^#^	0.62 ± 0.32 ^#^	0
Capsule	0.87 ± 0.35 ^#^	0.87 ± 0.35 ^#^	0

## Data Availability

The experimental data used to support the findings of this study are included within the article.
